# Reduction of Streptolysin O (SLO) Pore-Forming Activity Enhances Inflammasome Activation

**DOI:** 10.3390/toxins5061105

**Published:** 2013-06-06

**Authors:** Peter A. Keyel, Robyn Roth, Wayne M. Yokoyama, John E. Heuser, Russell D. Salter

**Affiliations:** 1 Department of Immunology, University of Pittsburgh, Pittsburgh, PA 15260, USA; E-Mail: pak55@pitt.edu; 2 Department of Cell Biology and Physiology, Washington University, St. Louis, MO 63110, USA; E-Mails: rroth22@wustl.edu (R.R.); jheuser@wustl.edu (J.E.H.); 3 Howard Hughes Medical Institute and Rheumatology Division, Department of Medicine, Washington University, St. Louis, MO 63110, USA; E-Mail: yokoyama@dom.wustl.edu

**Keywords:** NLRP3, inflammasome, Caspase-1, plasma membrane repair, streptolysin O

## Abstract

Pore-forming toxins are utilized by bacterial and mammalian cells to exert pathogenic effects and induce cell lysis. In addition to rapid plasma membrane repair, macrophages respond to pore-forming toxins through activation of the NLRP3 inflammasome, leading to IL-1β secretion and pyroptosis. The structural determinants of pore-forming toxins required for NLRP3 activation remain unknown. Here, we demonstrate using streptolysin O (SLO) that pore-formation controls IL-1β secretion and direct toxicity. An SLO mutant incapable of pore-formation did not promote direct killing, pyroptosis or IL-1β production. This indicated that pore formation is necessary for inflammasome activation. However, a partially active mutant (SLO N402C) that was less toxic to macrophages than wild-type SLO, even at concentrations that directly lysed an equivalent number of red blood cells, enhanced IL-1β production but did not alter pyroptosis. This suggests that direct lysis may attenuate immune responses by preventing macrophages from successfully repairing their plasma membrane and elaborating more robust cytokine production. We suggest that mutagenesis of pore-forming toxins represents a strategy to enhance adjuvant activity.

## 1. Introduction

Many strains of bacteria secrete pore-forming toxins (PFTs) that assist in their pathogenesis and survival. The best-studied PFT is the cholesterol-dependent cytolysin (CDC) secreted by the Group A Streptococcus, *Streptococcus pyogenes*, termed streptolysin O (SLO). SLO acts as a virulence factor [1], potentially due to its ability to facilitate the introduction of other bacterial products into cells [2,3] via cytosol-mediated translocation. Structurally, SLO is produced as monomer containing four domains, similar to other CDCs. Domains 1 and 3 together correspond to the MACPF domain found in the mammalian PFTs perforin and the complement membrane attack complex [4]. Domain 4 contains an undecapeptide which binds to cholesterol. After binding to cholesterol, 35–50 CDC monomers oligomerize into ring-shaped prepores 30 nm wide on the membrane [5,6]. Once this prepore is assembled, the domains undergo a coordinated conformation change from alpha helices to insert two beta sheets into the membrane, forming a beta-barrel structure pore in the membrane [7,8]. 

The structure of SLO has been well-mapped through mutagenesis studies and scanning cysteine mutations [9,10,11]. A single Cys at position 530 (C530) confers oxidative sensitivity to SLO. Mutation of C530 to Ala relieves the oxidative sensitivity without compromising pore-forming function [12]. This mutant has been used for cysteine scanning mutagenesis, allowing structure-function mapping of SLO [9,10,11]. In the context of the C530A mutation, further mutation of residue Asn 402 (N402) in domain 3 to Cys reduces toxicity to 10% of C530A without altering membrane binding [9,13]. EM of SLO C530A/N402C bound to cholesterol crystals reveals that in addition to pores, SLO C530A/N402C forms numerous linear arrays, arcs and nearly complete rings on the crystals, providing a mechanistic basis for impaired toxicity [13]. Derivatization of C530A/N402C with fluorescein maleimide completely abrogates toxicity, as does mutation of N402 to Glu and eliminates curvature in oligomerized SLO [9,13]. What effects these mutations have on the pathogenesis of *S. pyogenes* or immune response to *S. pyogenes* remains unknown. 

Mammalian cells recognize and respond to *S. pyogenes* in multiple ways. Metabolically active cells resist PFTs through the process of plasma membrane repair, whereby they seal off damaged portions of the plasma membrane [14]. In response to SLO, cells sequester the toxin on blebs and subsequently shed the blebs to remove the toxin from the cell [15]. In addition to cell survival mechanisms, when macrophages are challenged with CDCs, they initiate responses that could potentially aid in immunity, including NO production, CD14 and IL-6 receptor shedding, and TLR signaling among others [16,17,18,19,20]. Importantly, CDCs including SLO and the similar tetanolysin O trigger activation of the NLRP3 inflammasome [21,22]. The NLRP3 inflammasome is a multi-protein complex that promotes inflammation. It is comprised of three proteins, a sensory NLR, the adaptor ASC and effector Caspase-1 (Casp1). Each sensory NLR detects one or more pathogen or danger associated molecular patterns. NLRP3 is a sensory NLR that detects a wide variety of these patterns, ranging from SLO, other PFTs and the potassium ionophore nigericin to alum and silica [23]. Following detection, NLRP3 activates and recruits ASC, which in turn oligomerizes Casp1, leading to Casp1 activation and autoproteolysis [24]. Casp1 then facilitates cleavage of pro-IL-1β and pro-IL-18 and secretion of their mature forms along with other proteins [25,26]. Casp1 also facilitates inflammatory cell death, termed pyroptosis [26,27]. Precisely how NLRP3 senses SLO remains unknown, though it is possible either potassium efflux or other stress resulting from membrane damage leads to NLRP3 activation [28,29]. Although *S. pyogenes* requires SLO to activate the NLRP3 inflammasome [21], it has not been determined whether pore formation is required nor what effects partially active mutants have on inflammasome activation and IL-1β secretion. 

In this study, we explored the requirement of SLO pore-formation for inflammasome activation and pyroptosis. We found that pore formation was necessary for IL-1β release and pyroptosis. However, the partially active SLO N402C mutation, which induces enlarged pores and linear strands, was not as directly toxic as SLO that forms wild type pores. Strikingly, introduction of N402C into SLO did not alter the extent of pyroptosis, though introduction of N402C into SLO induced increased IL-1β production. This increase was due to additional mature IL-1β secreted by the macrophages. Taken together, these results indicate that less toxic SLO variants that produce strands and altered pores could promote a more robust immune response through enhanced cytokine secretion. 

## 2. Results

### 2.1. Altered Pore Formation in SLO N402C Mutant

**Figure 1:**
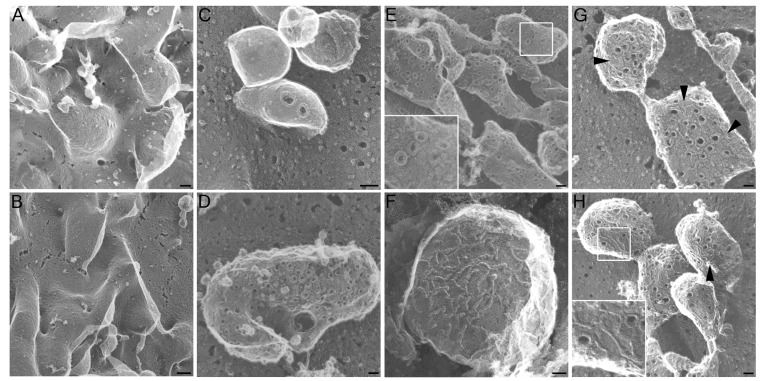
**Pore formation of streptolysin O (SLO) mutants**. Blebbing was induced (**A**,**B**,**D**–**G**), or not induced (**C**) in CHO cells by treatment with 25 mM paraformaldehyde and 2 mM DTT. Subsequently, no SLO (**A**), 10,000 U/mL SLO C530A (**B**,**D**,**E**) or an equivalent mass of SLO N402E (**F**) or SLO N402C (**G**,**H**) were added to the cells, and the cells were prepared for EM. Alternatively, living CHO cells were treated with 125 U/mL SLO for 5 min (**C**) and prepared for EM. Arrowheads indicate strands formed by SLO N402C. Scale bar = 1 μm (**A**,**B**) or 50 nm (**C**–**H**).

We investigated the ability of SLO mutants to form pores in our system. To reduce effects of SLO-induced plasma membrane blebbing to better visualize the toxins, we induced cellular blebbing in CHO cells using paraformaldehyde and DTT as previously described [30], prior to SLO challenge (Figure 1A). Addition of SLO C530A did not grossly alter the induced blebs (Figure 1B). As we have previously observed for SLO [15], wild type SLO forms pores with an outer diameter measuring 30 nm across in living cells (Figure 1C) and in previously fixed cells [15]. On CHO cells treated with paraformaldehyde and DTT, SLO C530A formed identical pores, indicating that the C530A mutation does not alter pore size, shape, or recruitment to blebs on the plasma membrane (Figure 1D,E), consistent with previous reports indicating that this mutation does not compromise toxin function [11,12,13]. Although the SLO N402E mutant, which lacks appreciable hemolytic activity [9], did localize to similar blebs, the ultrastructure of the assembled protein complex was radically different. SLO N402E did not form pores, instead forming linear arrays (Figure 1F). Interestingly, we found that on mammalian cells the partially active SLO N402C formed strands and partially complete pores (Figure 1G,H, arrowheads), similar to observations of SLO C530A/N402C on cholesterol microcrystals [13]. However, we did observe intact pores which were larger and more heterogeneous than native SLO pores (Figure 1G). The inner diameter of SLO C530A pores measured 23.2 ± 5.2 nm whereas the inner diameter of complete SLO N402C pores measured 44.5 ± 7.9 nm. Taken together, these data provide a structural basis for the reduced hemolytic activity of the SLO N402C and absent hemolytic activity of the SLO N402E mutant.

### 2.2. SLO N402C Has Reduced Toxicity in Mammalian Cells

**Figure 2:**
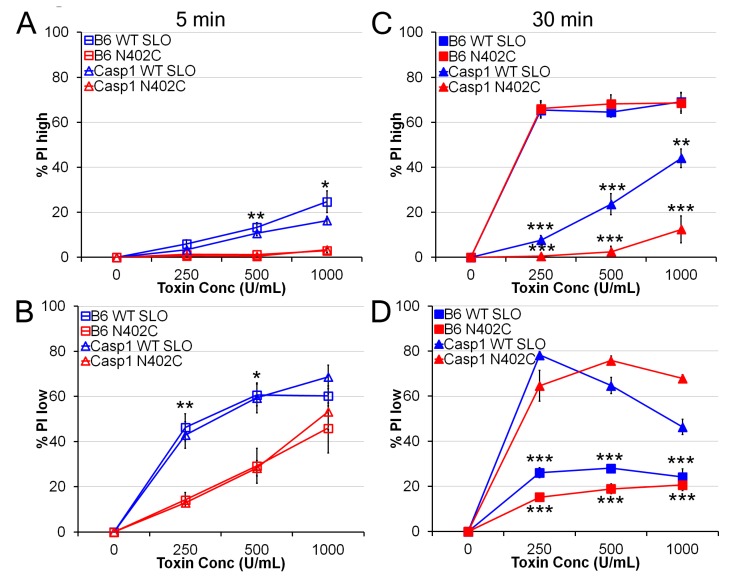
**SLO N402C shows reduced direct toxicity**. LPS-primed bone marrow-derived macrophages (BMDM)from B6 or Casp1^−/−^ mice were treated with the indicated SLO mutants at the indicated concentrations for 5 min (**A**,**B**) or 30 min (**C**,**D**) and PI uptake analyzed by FACS. The percentage of PI^high^, or dead cells (**A**,**C**) or percentage of PI^l^°^w^, or transiently permeabilized cells (**B**,**D**) are shown. The graphs display the average ± SEM of 3 independent experiments. * indicates *p <* 0.05, ** indicates *p <* 0.01 and *** indicates *p <* 0.001 for comparisons between wild type and N402C SLO (**A**,**B**) or comparisons between B6 and Casp1^−/−^ BMDM (**C**,**D**).

We next asked whether SLO N402C and C530A/N402C was as toxic to mammalian cells as wild type SLO pores. The N402C mutation reduced the hemolytic activity of SLO by 90%, so we normalized the concentration to equivalent hemolytic units of toxin. We treated LPS-primed bone marrow-derived macrophages (BMDM) with a range of SLO concentrations for 5 min at 37 °C and assessed propidium iodide (PI) uptake. Cells that display a 3-log shift by FACS are dead cells (PI^high^), whereas cells that only display a 1-log shift are transiently permeabilized (PI^l^°^w^) [15]. Using similar hemolytic activities between SLO and SLO N402C, we observed reduced killing by SLO N402C (Figure 2A). There was a decrease in killing at 1000 U/mL and 500 U/mL (Figure 2A). In contrast, the SLO N402E mutant, which only forms strands, had no cytotoxic activity even at a mass equivalent to the highest concentration of the SLO N402C used (data not shown). We observed an increase in transiently permeabilized BMDM treated with SLO N402C over those treated with SLO C530A (Figure 2B). This indicates that SLO N402C did form pores in the membrane of the BMDM, but the BMDM successfully resealed and survived the initial toxin attack. Taken together, we conclude that SLO N402C is not as toxic as SLO, which we ascribe to impaired pore-formation.

Given the difference in direct toxicity, we next asked whether pyroptosis was impaired in BMDM. In non-immune cells like 3T3 or CHO, SLO toxicity is similar at both 5 and 30 min [15]. In BMDM with active inflammasomes, however, Casp1 and Casp11 induce pyroptosis, which is evident by 30 min [27,31,32]. Generation of Casp1^−/−^ mice was done using 129 mice, which also lack functional Casp11 [32], and we will refer to cells generated from these mice as Casp1^−/−^ BMDM. When Casp1^−/−^ BMDM were treated with SLO or SLO N402C for 5 min, we observed no difference in either toxicity or permeability (Figure 2A,B). However, at 30 min, Casp1^−/−^ BMDM showed very little cell death (Figure 2C). These cells were transiently permeabilized, as indicated by the low levels of PI uptake (Figure 2D). In contrast, concentrations of SLO that induced <20% cell death at 5 min led to >60% death by 30 min (Figure 2A,C). Thus, comparing cell death at 5 min and 30 min reveals pyroptotic activity.

We next compared the ability of SLO mutants to induce pyroptosis in LPS-primed BMDM. Strikingly, SLO N402C caused equivalent levels of cell death to SLO at 30 min (Figure 2C), suggesting that pyroptosis was equivalent between wild type and N402C SLO. The percentage of transiently permeabilized cells was reduced following treatment with both mutants, indicating that the population that successfully resealed likely executed pyroptosis (Figure 2D). This suggested that BMDM can activate the inflammasome even after successfully repairing their membranes. Notably, SLO N402E did not trigger pyroptosis, indicating that SLO pore formation is necessary for the induction of pyroptosis (data not shown). We conclude that BMDM respond as aggressively to partially toxic proteins as they do to highly toxic ones.

### 2.3. SLO C530A/N402C Has Similarly Reduced Toxicity

**Figure 3:**
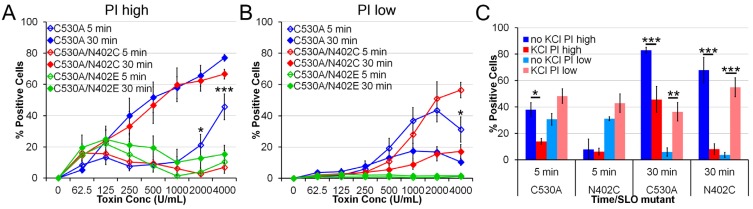
**SLO C530A/N402C also shows reduced toxicity.** LPS-primed BMDM were treated with the indicated SLO mutants at the indicated concentrations (1000 U/mL for **C**) for 5 min or 30 min in the absence (**A**–**C**) or presence (**C**) of 50 mM KCl and PI uptake analyzed by FACS. The percentage of PI^high^, or dead cells (**A**,**C**) or percentage of PI^l^°^w^, or transiently permeabilized cells (**B**,**C**) are shown. The graphs display the average ± SEM of 3 independent experiments. * indicates *p <* 0.05, ** indicates *p <* 0.01 and *** indicates *p <* 0.001.

Since we observed a difference in pyroptosis between wild type and N402C SLO, we next asked whether this difference also occurred between C530A and C530A/N402C SLO. Given the redox sensitivity of wild type SLO, the redox-insensitive C530A mutation would be necessary for stability in any adjuvant trials. We treated LPS-primed BMDM with a range of concentrations of SLO C530A, C530A/N402C and C530A/N402E and measured both direct toxicity and pyroptosis. Similarly to SLO N402C, C530A/N402C showed impaired toxicity at 5 min with a concomitant increase in transiently permeabilized cells was observed (Figure 3A,B). Furthermore, pyroptosis was not impaired between C530A and C530A/N402C SLO mutants (Figure 3A). Importantly, SLO C530A/N402E did not induce cell death, consistent with its inability to form pores. Taken together, we conclude that introduction of C530A mutation does not alter the ability of N402C to impair direct toxicity without altering pyroptosis.

To further explore the relationship between cell death in our system and inflammasome activation, we treated LPS-primed BMDM with KCl, an inhibitor of the NLRP3 inflammasome, and measured cell permeabilization and death. We found that KCl treatment partially reduced direct lysis by SLO C530A though it did not significantly increase the PI^l^°^w^ population (Figure 3C). Importantly, KCl reduced pyroptosis down to levels observed due to direct lysis of BMDM following treatment with both SLO C530A and SLO C530A/N402C (Figure 3C). In place of the lysis, we observed an increase in the number of transiently permeabilized cells (Figure 3C). This validates that these cells were permeabilized, but prevented from executing a NLRP3-dependent pyroptotic program. We observed similar results with SLO and N402C mutations (data not shown). We conclude that SLO C530A/N402C induces increased pyroptosis over SLO C530A.

### 2.4. Increased IL-1β Production by SLO N402C

**Figure 4:**
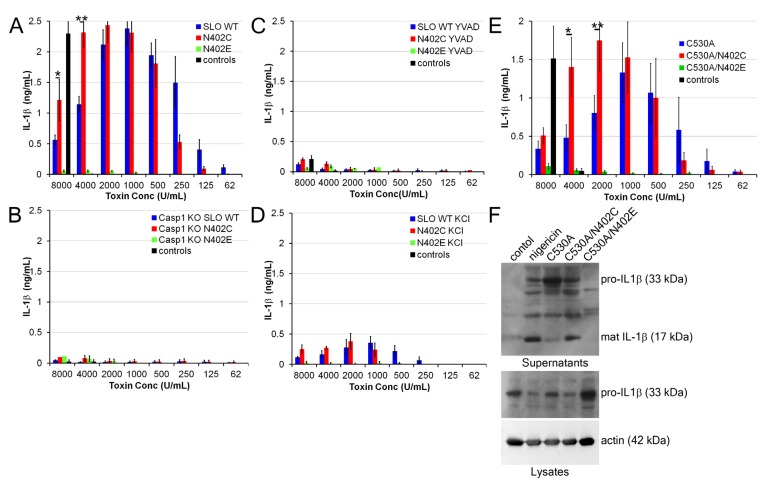
**SLO N402C enhances IL-1β release**. (**A**–**E**) LPS-primed B6 or Casp1^−/−^ BMDM were treated with the indicated inhibitors and concentrations of SLO mutants for 30 min, or the controls nigericin or nothing. Supernatants were harvested and assayed for IL-1β levels by ELISA. (**F**) LPS-primed BMDM were treated as indicated with controls or 2000 U/mL of each SLO mutant for 30 min. Supernatants were collected and TCA precipitated, while cells were lysed in SDS-sample buffer. Lysates and supernatants were resolved by SDS-PAGE, transferred to PVDF and probed with anti-IL-1β monoclonal antibody 3ZD and anti-actin monoclonal antibody. The graphs display the average of 4 independent experiments ± SEM, while the blot shows one representative experiment of 5. * indicates *p <* 0.05 and ** indicates *p <* 0.01.

Since we observed a difference in pyroptosis, we next asked whether the processing and secretion of IL-1β was altered by SLO N402C. We treated LPS-primed BMDM with a range of SLO concentrations and measured IL-1β levels in the supernatant 30 min after challenge. Importantly, we had observed equivalent cell death at these time points (Figure 2C). We found that SLO induced IL-1β in a dose-dependent manner (Figure 4A), starting at concentrations similar to those that induced pyroptosis. However, once direct toxicity of SLO started to increase, the amount of IL-1β decreased (Figure 4A). Interestingly, the SLO N402C mutant showed an increased range over which IL-1β was secreted and induced higher maximal levels of IL-1β secretion compared to the SLO (Figure 4A). Importantly, the SLO N402E mutant did not promote IL-1β production (Figure 4A). These data indicate that a similar hemolytic activity of SLO N402C promotes greater IL-1β secretion than wild type SLO.

IL-1β secretion triggered by pore-forming toxins is dependent on the NLRP3 inflammasome [21,22]. To demonstrate that both SLO and SLO N402C were triggering inflammasome-dependent IL-1β secretion, we treated LPS-primed Casp1^−/−^ BMDM with SLO or SLO N402C and measured IL-1β secretion by ELISA. We found no IL-1β secretion under these circumstances (Figure 4B). Similarly, treatment with the Casp1 inhibitor YVAD blocked IL-1β release, as did KCl (Figure 4C,D). Taken together, these data indicate that the NLRP3 inflammasome is necessary for both wild type and mutant SLO.

We next compared the effect of introducing the C530A mutation into SLO on IL-1β production. As observed for wild type SLO, we found an increase in IL-1β production by SLO C530A/N402C over that produced by SLO C530A at higher concentrations (Figure 4E). This further indicates that the C530A mutation does not adversely affect SLO. 

We next determined whether the increase in IL-1β secretion was due to the secretion of pro-IL-1β or mature IL-1β. We examined cell lysates and supernatants by immunoblot, and found that after 30 min with 2000 U/mL of toxin, SLO C530A promoted a mixture of pro- and mature IL-1β secretion, though predominantly pro-IL-1β (Figure 4F). This corresponds to the trailing levels of IL-1β detected by ELISA and increased direct toxicity observed in BMDM at this concentration. In contrast, SLO C530A/N402C displayed high levels of mature IL-1β and low levels of pro-IL-1β in the supernatants under similar circumstances (Figure 4F). Taken together, these data indicate that SLO C530A/N402C increases the amount of mature IL-1β secreted by BMDM.

## 3. Discussion

Here we have examined the structure-function of SLO pore formation in inflammasome activation. We show that pore formation by SLO is required for inflammasome activation. We further demonstrate that a mutant that forms abnormal pores is less lethal to macrophages, allowing them to produce increased amounts of IL-1β. Taken together, our data suggest that optimization of IL-1β production requires that macrophage survive the membrane disruption that initiates NLRP3 stimulation.

We find that the SLO N402C appeared similar on mammalian cell membranes to what was previously observed using SLO C530A/N402C on cholesterol crystals [13]. These images reveal a mechanistic basis for the pore-formation defect in the SLO N402C: impaired ring formation. Importantly, as previously described [9], there is no defect in membrane binding with this mutant. Mutation of N402C does not completely abrogate pore formation, as wider pores were formed, though the N402E mutation prevented pore formation. These wider pores have a variety of biological consequences when they interact with immune cells.

To rule out decreased responses due to the impaired hemolytic ability of SLO N402C, we used an equivalent hemolytic dose of toxin, which typically corresponded to a 10-fold increase in protein concentration. Even at this concentration, we observed decreased direct killing of the macrophages by SLO N402C compared to wild-type SLO. This suggests to us that even at an equivalent hemolytic dose, the larger pores and strands are more readily resisted by macrophage membrane repair processes. Rapid polymerization of PFTs may be necessary to successfully intoxicate cells and overcame active membrane repair responses. 

Despite the increased ability of macrophages to resist direct lysis by SLO N402C, we observed no difference in the extent of cell death 30 min later, at which point in time transiently permeabilized macrophages have undergone pyroptosis. This indicates that the membrane repair response does not alter the intracellular signaling events (*i.e.*, pyroptosis), even when membrane repair reduced direct lysis. Similar results have been observed with the immune PFT perforin, where decreasing direct lysis of cells by modulating calcium concentration did not alter the ability of cells to externalize phosphatidylserine or receive granzyme B [33]. We propose that membrane repair responses are protective, and bacterial toxins have evolved to provide rapid and effective lysis of target cells. Mammalian immune cells such as natural killer and CD8+ T cells have utilized a secondary toxin approach, where granzyme delivery ensures death of those cells that survived the initial perforin hit. 

In contrast to hypotheses that membrane repair is subverted by PFT attack [34], we propose that membrane repair is a cellular defense against PFTs, and observed downstream signaling events (such as inflammasome activation) following PFT attack are instead responses to the transient permeabilization experienced by cells. This proposal is consistent with the finding that many NLRP3 agonists promote membrane disruption [28,29]. Furthermore, for those NLRP3 agonists which cause membrane damage, it has not been possible to separate the membrane-damaging aspect from successful NLRP3 activation. We show that pore formation is required for SLO to act as an NLRP3 agonist. Additionally, we observe transient permeabilization in macrophages when pyroptosis is blocked, either using Casp1^−/−^ BMDM or KCl, suggesting that it is this population that proceeds to execute pyroptosis. When we treated macrophages with KCl or used Casp1^−/−^ BMDM, we found an increase in the transiently permeabilized population in place of the pyroptotic population. We also observed an increase in the amount of IL-1β secreted in the context of the less damaging, but equally hemolytic, N402C mutant. This was due to an increase in the production of mature IL-1β. We attribute the increased capacity for IL-1β production to an increase in the number of cells that survived initial contact with SLO. 

In addition to mechanistic insights to PFT biology, these findings have practical implications as well. The only currently FDA-approved adjuvant, alum, activates the NLRP3 inflammasome [35,36,37], suggesting other NLRP3 agonists could serve as potential adjuvants. SLO has several advantages over other NLRP3 agonists. Unlike those agonists that induce lysosomal disruption, SLO acts at the surface. SLO is also titratable over a range of concentrations, allowing fine-tuning of the extent of adjuvant effect. Extensive mutagenesis studies have been performed on SLO [9,10,11], enabling researchers to control various aspects of toxin function. We show here that enlarging the pores and decreasing toxicity, for example, both lead to increased IL-1β production. These results indicate that SLO mutants show promise as adjuvants.

## 4. Experimental Section

### 4.1. Reagents

Unless otherwise specified, all reagents were from Fisher (Waltham, MA). IL-1β ELISA antibodies were from eBioscience (San Diego, CA, USA), anti-IL-1β 3ZD monoclonal antibody was from the Frederick National Laboratory for Cancer Research (Frederick, MD, USA), anti-actin AC-15 monoclonal antibody was from Sigma (St Louis, MO, USA) and anti-mouse antibodies conjugated to HRP were from Jackson Immunoresearch (West Grove, PA, USA). SLO mutants were constructed using QuikChange Mutagenesis (Agilent Technologies, Santa Clara, CA, USA) to introduce C530A, N402C and N402E mutations into wild type SLO [38]. SLO was purified as previously described [39]. The hemolytic activity was defined as the amount of toxin needed to lyse 50% of a 2.5% solution of sheep RBCs in 30 min at 37 °C. It was typically 2.4 × 10^6^ U/mg, 2.5 × 10^5^ U/mg, 531 U/mg, 4.9 × 10^5^ U/mg, 4.6 × 10^4^ U/mg and 68 U/mg for SLO wild type, N402C, N402E, C530A, C530A/N402C and C530A/N402E, respectively.

### 4.2. Cell Culture

CHO cells were cultured in F12 medium supplemented with 10% FCS and 1× l-glutamine. Primary macrophages were isolated from C57BL/6 or Casp1^−/−^ mouse bone marrow as previously described [22]. BMDM were differentiated for 7–21 days in DMEM supplemented with 30% L929 cell supernatants, 20% FCS, 1× sodium pyruvate, 1× l-glutamine and 1× penicillin/streptomycin. 

### 4.3. Electron Microscopy

Rapid-freeze/deep-etch electron microscopy was performed as previously described [15]. CHO cells were induced to generate blebs as previously described [30] using 25 mM paraformaldehyde and 2 mM DTT for 15 min, washed three times in Ringer’s solution, treated with 10,000 U/mL SLO C530A or equivalent mass of SLO N402C or SLO N402E at 37 °C for 5 min, washed three times in Ringer’s solution, fixed in 2% glutaraldehyde and prepared for electron microscopy. Alternatively, CHO cells were treated with 125 U/mL SLO for 5 min prior to washing and glutaraldehyde fixation. Platinum replicas were prepared as described [40], examined in a 100CX microscope (JEOL, Peabody, MA, USA) and imaged with an AMT digital camera (Danvers, MA, USA). Inner pore diameters were determined by measuring at least 30 pores viewed at 50,000× using Photoshop (Adobe, San Jose, CA, USA). To accurately determine the length of pores on curved membranes, the diameter parallel to the curved portion of the membrane was used.

### 4.4. Toxicity Assay

BMDM were primed with 50 ng/mL LPS (Invivogen, San Diego, CA, USA) for 4 H at 37 °C and harvested with Cellstripper. BMDM were resuspended in RPMI containing 40 μg/mL PI in 96-well V-bottom plates. SLO mutants were diluted to 2× in RPMI and added to the BMDM in the presence of absence of 50 mM KCl for either 5 min or 30 min at 37 °C. BMDM were harvested by pipetting and examined by flow cytometry on a Fortessa (BD Biosciences, San Jose, CA, USA). The percent of PI+ cell was determined with the following formula: %PI = (%PI_exp_ − %PI_control_)/(100-%PI_con__trol_). Cells that showed a 3-log shift in PI fluorescence (PI^high^ cells) correspond to dead cells, while those that show a 1-log shift in PI fluorescence (PI^l^°^w^ cells) correspond to transiently permeabilized cells [15]. 

### 4.5. IL-1β Release

BMDM were plated at either 10^5^ cells/24-well (ELISA) or 10^6^ cells/6-well (western blot), primed with 10 ng/mL LPS for 4 H at 37 °C, treated with 50 mM KCl or 100 μM YVAD for 30 min and challenged with the indicated concentrations of SLO mutants or 20 μM nigericin (Sigma) for 30 min at 37 °C. For ELISA, supernatants were collected and analyzed as previously described [22]. For western blots, supernatants were TCA-precipitated as previously described [22], while cells were directly lysed with 95 °C 1× SDS-sample buffer and sonicated. One tenth of cell lysates or one-half of cell supernatants were resolved by SDS-PAGE using a 12.5% gel, transferred to PVDF, probed with 3ZD anti-IL-1β monoclonal antibody, anti-mouse-HRP secondary antibody, and visualized with enhanced chemiluminescent reagent (Santa Cruz Biotechnologies, Santa Cruz, CA, USA). Blots were stripped and reprobed with AC-15 anti-actin monoclonal antibody and anti-mouse-HRP secondary antibody.

### 4.6. Statistics

Significance was determined by two-way ANOVA with Bonferroni post-testing using Prism 3.0 (Graphpad, La Jolla, CA, USA).

## 5. Conclusions

In this study, we have explored the structure-function of the CDC streptolysin O. We found that mutation of N402C impairs pore formation by inducing strand formation and enlarged rings. This impairment of pore formation has biological consequences, as equivalent hemolytic units of SLO and SLO N402C do not have equivalent toxicity in macrophages. We show increased cell survival in SLO N402C, and suggest this could be due to reduced damage or more robust membrane repair due to the larger pores and strands. The transient permeabilization, however, still activated downstream intracellular signaling cascades. In the case of macrophages, those processes were IL-1β production and secretion along with pyroptosis. Due to the increased viability of macrophages following SLO challenge, we observed an increase in the amount of IL-1β produced by the macrophages. This suggests that direct toxicity of PFTs helps bacteria by blocking immune responses, and provides insight for future vaccine development.
